# Resection Cavity Contraction Effects in the Use of Radioactive Sources (1-25 versus Cs-131) for Intra-Operative Brain Implants

**DOI:** 10.7759/cureus.2079

**Published:** 2018-01-16

**Authors:** Dae Y Han, Lijun Ma, Steve Braunstein, David Raleigh, Patricia K Sneed, Michael McDermott

**Affiliations:** 1 Department of Radiation Oncology, University of California, San Francisco; 2 Department of Neurological Surgery, University of California, San Francisco

**Keywords:** brain metastasis, brachytherapy, resection cavity, ldr, cs-131, i-125

## Abstract

Background and Objectives

Intra-parenchymal brain surgical resection cavities usually contract in volume following low dose rate (LDR) brachytherapy implants. In this study, we systematically modeled and assessed dose variability resulting from such changes for I-125 versus Cs-131 radioactive sources.

Methods

Resection cavity contraction was modeled based on 95 consecutive patient cases, using surveillance magnetic resonance (MR) images. The model was derived for single point source geometry and then fully simulated in 3D where I-125 or Cs-131 seeds were placed on the surface of an ellipsoidal resection cavity. Dose distribution estimated via TG-43 calculations and biological effective dose (BED) calculations were compared for both I-125 and Cs-131, accounting for resection cavity contractions.

Results

Resection cavity volumes were found to contract with an effective half-life of approximately 3.4 months (time to reach 50% of maximum volume contraction). As a result, significant differences in dose distributions were noted between I-125 and Cs-131 radioactive sources. For example, when comparing with static volume, assuming no contraction effect, I-125 exhibited a 31.8% and 30.5% increase in D90 and D10 values (i.e., the minimal dose to 90% and 10% of the volume respectively) in the peripheral target areas over the follow-up period of 20.5 months. In contrast, Cs-131 seeds only exhibited a 1.44% and 0.64% increase in D90 and D10 values respectively. Such discrepancy is likewise similar for BED calculations.

Conclusion

Resection cavity contractions affects Cs-131 dose distribution significantly less than that of I-125 for permanent brain implants. Care must be taken to account for cavity contractions when prescribing accumulative doses of a radioactive source in performing the brain implant procedures.

## Introduction

Brain metastases are the most common intracranial malignancy. Intraoperative permanent brain implants have been performed since the 1980s for treating large brain metastases [1]. The procedure aims to combine surgery and radiation therapy in a single procedure in order to resolve mass effects and related symptoms in a timely manner [1-4]. Historically, I-125 has been our radioactive source of choice for performing permanent brain implants [2-4]. I-125 possesses a half-life of 60 days and emits photons with a mean energy of approximately 0.3 MeV. Lately, however, Cs-131 has emerged as an alternative for permanent brain implants [5-6]. Cs-131 possesses a half-life of 9.7 days and emits photons that reach peak energies of 0.29 to 0.34 MeV [7-9].

We have analyzed our institution’s experience of using I-125 brain implants for treating large brain metastases [4]. In particular, the resection cavity volume was systematically examined on surveillance magnetic resonance (MR) imaging. Significant changes in the resection volume were found over the follow-up period of one to two years, with as much as 60% volume contraction noted postoperatively.   

The question, therefore, arises as to how to correlate and compare I-125 based brain implants with the Cs-131 based procedure in the context of continuous resection cavity volume changes. To address this question, we first developed an empirical dynamic target volume (DTV) model to quantify observed resection cavity volume changes. We then investigated such an effect via theoretical dose calculations with point source approximations. Finally, we performed detailed brute-force TG43 calculations as well as BED calculations with the DTV model to evaluate and compare the 3D dose distributions of the I-125 and Cs-131 radioactive sources subject to resection cavity volume changes. The goal of our study is to provide a physical dose guided rationale for performing permanent brain implants with Cs-131 radioactive sources.

## Materials and methods

Patient data and empirical DTV modelling

The empirical DTV model was derived from 95 consecutive patients with 105 brain metastases treated at our institution from September 1997 to July 2013 [4]. All patients underwent a postoperative computed tomography (CT) scan for post-plan dosimetry and the data were analyzed via a commercial platform (MIM Vista, Cleveland, OH). The patients were followed with serial surveillance T1-weighted magnetic resonance imaging (MRI) scans at regular intervals, and the metastasis resection cavity volume data were determined retrospectively from these MR images.  

The median postoperative resection cavity volume was measured to be 5.2 mL (range: 0.3 - 23.2 mL). Permanent brain implants were performed for all cases using I-125 radioactive sources (Model 6711, GE Healthcare) at the time of surgical resection. The radioactive sources were placed evenly 6-10 mm apart, with 28 radioactive sources on average implanted per cavity. The initial source activity ranged from 0.3 mCi to 1.3 mCi [3-4].

Using ideal point source approximation, the dose rate at a given time t from a single source can be calculated as

                     

$$\dot{D_0}(t)=\dot{D_0}e^{-\lambda t}=C e^{-\lambda t}/r^{\epsilon} \; \; (1)$$

where C is a constant (i.e., C = S*Λ*$\phi$, S = initial air-kerma strength, Λ = dose rate constant, and $\phi$= mean anisotropy value), $\lambda$ = decay constant for the source (i.e., $\lambda$=0.693/T_1/2_ where T_1/2_ = half-life of the radioisotope).  

In Equation 1, we have approximated the radial dependence as a power function and the published values of power index $\epsilon$ = 2.2 for the Cs-131 (Model CS-1) and $\epsilon$ = 2.4  for the I-125 (Model 6711) sources, respectively [7]. Note that the near-source dose fall-off was slightly steeper for the point I-125 sources than the Cs-131 sources.

If no seed migration is assumed during the resection cavity contraction and its movement is isotropic, then the distance between a point in space to any radioactive source located on the surface of the resection cavity is given as follows:

                                       

$$r(t)\propto V(t)^{1/3}\; \; \; (2)$$

Therefore, Equation 1 can be rendered to Equation 3 as follows:

                      

$$\dot{D}(t)=\dot{D_0}e^{-\lambda t}/R_{0}\cdot (V(t)/V(t_0))^{-\epsilon /3}\equiv \dot{D_0}/R_0\cdot DCF(t) \; \; (3)$$

where $\dot D(t_0)$ denotes the initial dose rate and R_0_ is the distance from the initial source position to the point of interest (i.e., the initial distance before any cavity contractions). In Equation 3, we have further defined a dynamic dose rate correction function DCF(t) that governs the time dependence of the dose rate function, i.e., 

                          

$$DCF(t)=e^{-\lambda t}\cdot [V(t)/V(t_0)]^{-\epsilon /3} \; \;\; (4)$$

             

In this study, we further modeled contraction of the resection cavity volume V(t) as follows based on clinical data, i.e.,

                                          

$$V(t)=V(0)(A+Be^{-\mu t}) \; \; \; (5)$$

where A, B,$\mu$ are the empirically fitted parameters.  

From Equation 5, we finally obtained the analytical form the DCF(t) as follows:

                              

$$DCF(t)=e^{-\lambda t}(A+Be^{-\mu t})^{-\epsilon /3} \; \; \; (6)$$

The physical meaning of the fitting parameters of Equation 6 can be interpreted as follows: $\mu$ is the mean cavity contraction constant such that 0.693/$\mu$ = the effective half-life of the cavity contraction (i.e., the time required to achieve 50% of volume (initial volume – minimum volume) as observed). A is the asymptotic percentage volume after a sufficiently long follow-up time, and B is the factor that scales the instantaneous contraction rate of the target volume. Note that if $\mu$ is a sufficiently large value (i.e., for isotopes with a short half-life), then the second term in Equation 6 can be approximated as a constant thus rendering DCF(t) only governed by the radioactive decay of a source.  

**Figure 1:**
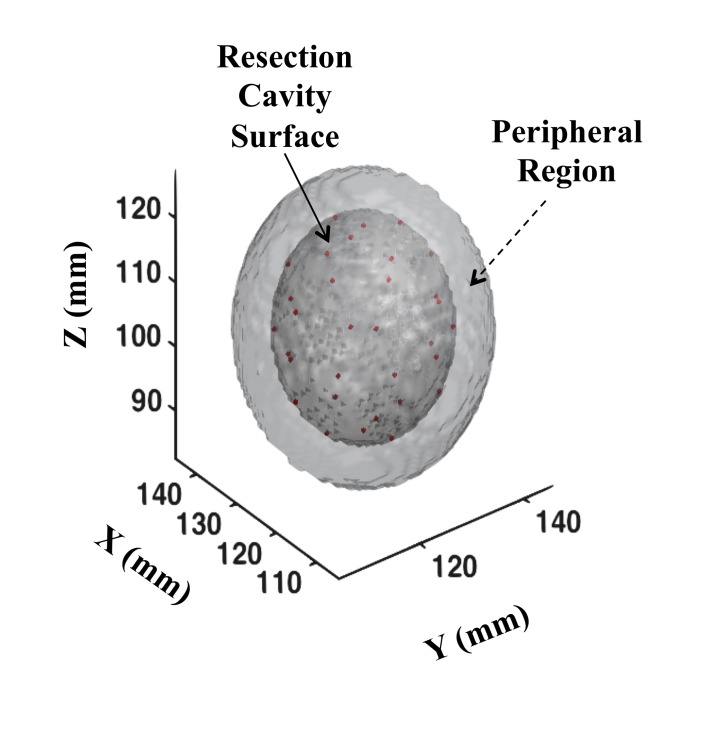
Three-dimensional plot of the resection cavity as modeled for the current study The radioactive sources (I-125 or Cs-131) are placed on the surface of the cavity and their center locations are denoted as red dots on the plotted surface.

To consider exact source geometry and anisotropy corrections, we further performed brute-force 3D calculations on simulated resection cavity geometry. The resection cavity was assumed to be an ellipsoid possessing a short radius of 1.4 cm and long radius of 1.6 cm for a total volume of 14.0 mL. Mimicking our clinical practices, 31 seeds (either all of them I-125 or Cs-131) were placed on the surface of the reconstructed volume every 7 mm along the longitudinal axis. Each cross-sectional plane thus possessed 1, 4, 6, or 10 seeds respectively (Figure 1). We further marked a 6-mm peripheral margin from the surface of the ellipsoid and designated it as the peripheral region of interest for the study. An isotropic contraction in the ellipsoid size was then applied to reflect the resection volume shrinkage per Equation 5. An in-house dose calculation engine [10] was implemented to perform 3D dose calculations via the updated TG-43 formalism [9, 11-12]. Of note, TG-43U1 data points were interpolated for 1×1×1 mm^3^ voxels encompassing a dose grid 10×10×10 cm^3^ in size. The final dose distribution at each voxel from multiple sources was determined as the sum of the individual source contributions via Equations 3-6. 

Dose and biological effective dose (BED) parameters

For 3D calculations, the half-life of I-125 is taken as 60.2 days, with 90% of the dose delivered at 204 days. Published parameters for the Model 6711 I-125 seeds were used [11-12]. The dose rate constant (Λ = 0.965 cGy/h/U), radial dose function g(r), and 2D anisotropy function (F(r,θ)) were referenced based on the TG-43 report [12]. The line source active length, L = 3.0 mm, was used for the geometry function (GL(r,θ)) calculations.

Published Cs-131 parameters for the Model CS-1 (model CS-1 Rev2, IsoRay, Richland, WA) were employed for our calculations [7,9]. The half-life of Cs-131 is 9.7 days and 90% of the dose is delivered in 33 days. The Cs-131 dosimetry parameters, including the dose rate constant (Λ = 1.046 cGy/h/U), radial dose function g(r), and 2D anisotropy function (F(r,θ)), were used [7,9]. The line source active length, L= 4.0 mm, was used for geometry function (GL(r,θ)) calculations.

The I-125 or Cs-131 seeds were arranged longitudinally (z-axis). Each source (I-125 and Cs-131) activities were properly decayed corresponding to the MR imaging time intervals in conjunction with the cavity contraction effect. The Cs-131 initial activity was also scaled to satisfy I-125 biological effect dose (BED) coverage for both implant procedures. In particular, D_90%_ and D_10%_ to the peripheral target region of interests were also calculated.

To calculate the biological effective dose (BED), a generic linear-quadratic (LQ) formula was adopted [13]. Since the BED calculation, in essence, applies a non-linear scaling factor to the physical dose values, the calculation itself does not affect the functional dependence of DCF of Equation 4. For simplicity, the following BED formula was adopted [12-13]:

                                       

$$BED=\frac{D}{\lambda } (1+\frac{D}{(\tau +\lambda )(\alpha /\beta )})\; \; \; (7)$$

where D is initial physical dose rate, and $\tau$  is the cell recovery constant ($\tau$ = ln(2)/T1/2 = ln(2)/1.5 = 0.462 (h^-1^) was employed for the current study). For the current study, we also adopted $\alpha/\beta$ = 2.1 Gy for the peripheral cavity region to assume that it is largely dominated by the normal brain in conjunction with the tumor cells. The rationale for us adopting such low $\alpha/\beta$ values was largely for the purpose of magnifying any differences in physical doses between the DTV and the static target volume implants. Evidently, the linear term would dominate Equation 7 for high $\alpha/\beta$ values while the quadratic term would dominate for low $\alpha/\beta$ values, thus making BED a sensitive tool to examine the overall effects of DTV on the brain implant dosimetry.

## Results

**Figure 2:**
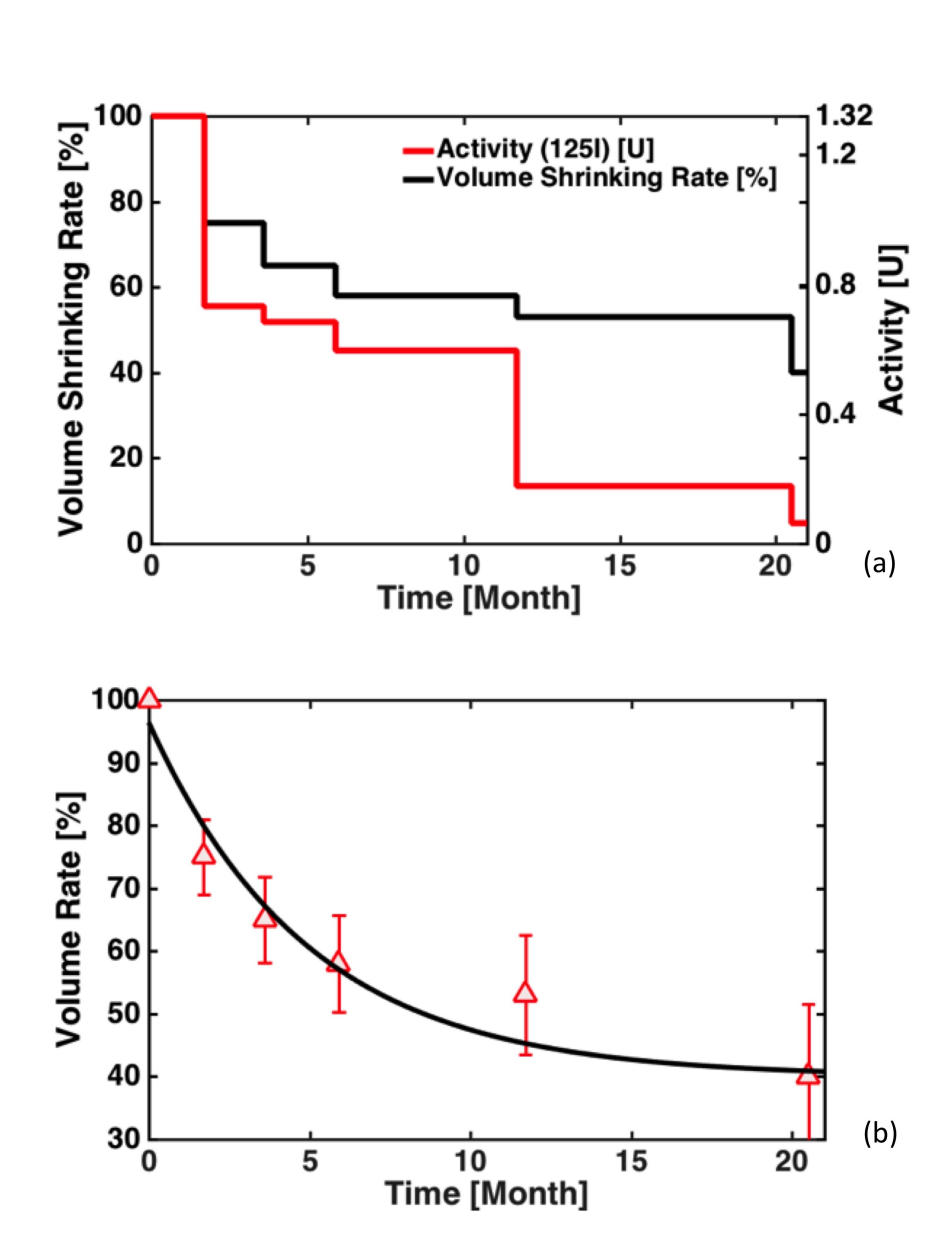
Dynamic target volume fitting results (a) Shows the percentage resection cavity volume contraction over a period of 20.5 months; (b) shows the fitted curve in the semi-logarithmic scale. Note that crossing of the curve at the origin was not constrained for the purpose of increasing overall integration accuracy of the curve.

A total of 476 MR images [4] were analyzed using surveillance MR scans performed at the median time intervals of 1.7, 3.6, 5.9, 11.7, and 20.5 months after resection. From the clinical data in Figure 2, the best fitting formula for DTV contraction as per Equation 5 was determined to be in the form of $V(t)/V(0)= 0.564&times;e^{-0.202t}+0.340$, where R^2 ^0.98 was obtained for curve fitting (Figure 2). From the fitted formula, we thus obtained that the effective half-life for the target volume contraction equals to 3.4 months. This is considerably longer than the decay half-life of Cs-131 (0.16 month) and somewhat longer than the decay half-life of I-125 (2.0 months). As predicted in the DCF expression of Equation 4, the second term of DCF can be negligible for Cs-131 while remaining significantly contributive for I-125 in brain implants.

**Figure 3:**
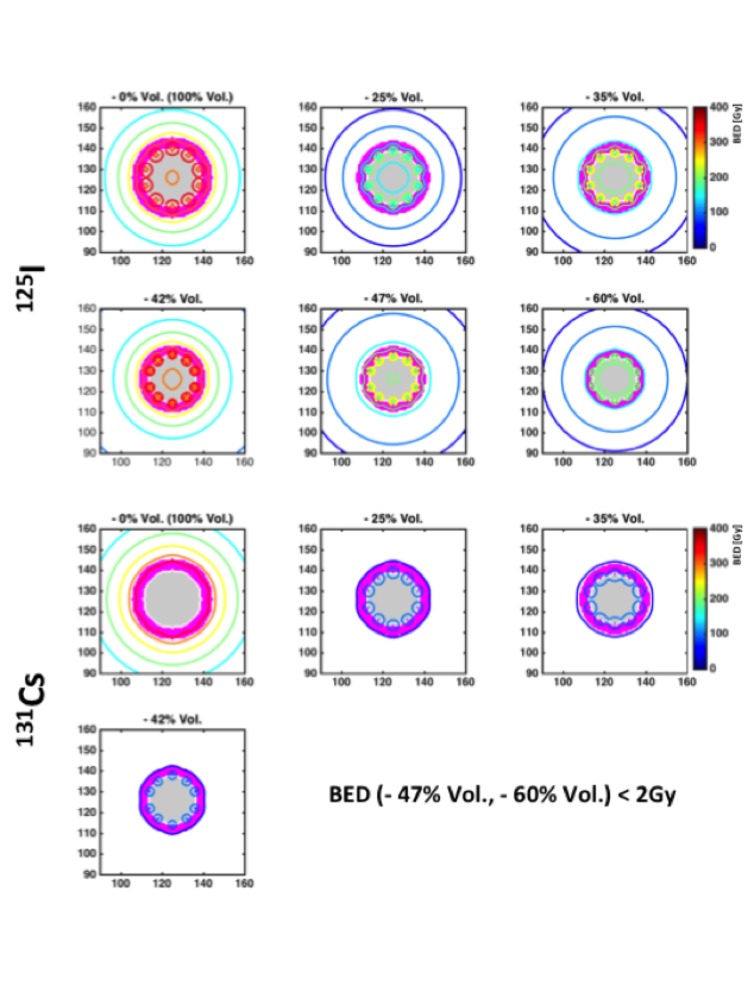
Comparison of the planar isodose distributions (within the transverse cross-sectional x-y planes) between I-125 and Cs-131 at 0, 1.7, 3.6, 5.9, 11.7, and 20.5 months after the implant procedure

The planar isodose distributions for both static and dynamic target volume contraction are compared in Figure 3. With the I-125 sources, the dose to the peripheral target region exhibited a 31.8% increase in the D_90%_ value (minimum dose to 90% volume of interest), and similarly, a 30.6% increase in the D_10%_ value due to the dynamic target volume contraction versus the static target volume. In contrast, only 1.4% increase in the D_90%_ and 0.6% increase in the D_10%_ value were observed for the Cs-131 seeds.

**Figure 4:**
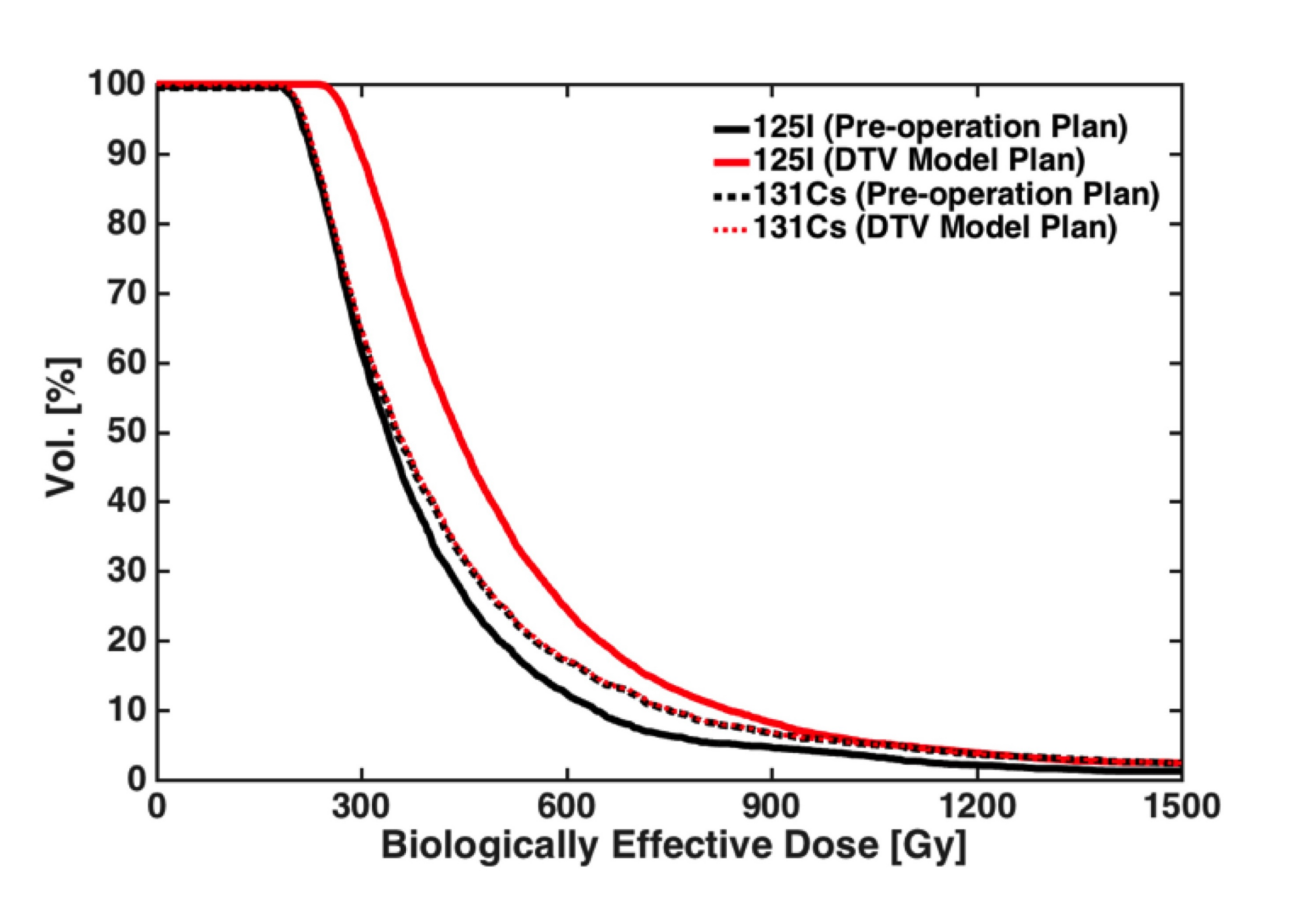
Comparison of the biological effective dose volume histograms (BEDVHs) between the pre-operation treatment plan assuming no target volume variations versus the dynamic target volume (DTV) treatment plan using either I-125 or Cs-131 radioactive sources.

The biological effective dose volume histogram (BEDVH) for dynamically contracting target volume (DTV) further confirmed the DCF prediction of Equation 4. In Figure 4, BEDVH for both I-125 and C-131 implants in the 3D elliposoidal case is given. Note that the initial source activities of I-125 and Cs-131 were scaled such that a nominal BED of 200 Gy was delivered to the same target volume coverage for both radioactive sources. With the introduction of the dynamic target volume effect, a negligible effect was noted for the Cs-131 in the BEDVH plotting. However, a large shift in the curve of approximately 25%-30% around 50% of the target volume region was noted along the x-axis for the I-125 implants (Figure 4). 

## Discussion

In light of resection cavity contractions post intraoperative brain brachytherapy, we have noted significant variations in the dose distributions for I-125 radioactive sources compared with the Cs-131 radioactive sources. As pointed out by the single source DCF formula, this was fundamentally caused by the fact that the effective half-life of the resection cavity contraction was observed to be 3.4 months. This is approximately 20 times that of the Cs-131 decay half-life of 0.16 months and approximately 1.7 times that of the I-125 decay half-life of 2.0 months. As a result, the target volume contraction negligibly affected the Cs-131 implant dosimetry while significantly influencing the I-125 implant dosimetry.

In terms of clinical impact, our data have shown that I-125 radioactive sources delivered approximately 30% more dose to the near target peripheral region when corrected for the resection cavity contraction effect. This implies that when replacing I-125 with Cs-131 radioisotopes for brain implant procedures, initial Cs-131 source activities should be carefully calibrated in order to match a biologically equivalent dose delivered to the target and to the surrounding normal tissue.

## Conclusions

Our study has shown that short half-life Cs-131 is significantly more robust than I-125 in response to resection cavity contraction effects for metastatic brain implant procedures. Clinical users should exercise caution when switching from one radioactive source to another with the intent to compare and correlate clinical experiences based on prior source protocols.
